# Anti-inflammatory activity of lauric acid, thiocolchicoside and thiocolchicoside-lauric acid formulation

**DOI:** 10.6026/973206300191075

**Published:** 2023-11-30

**Authors:** Ameena Mustafa, Meignana Arumugham Indiran, Rajeshkumar Shanmugham, Karthikeyan Ramalingam

**Affiliations:** 1 Department of Oral Pathology and Microbiology, Saveetha Dental College and Hospitals, Saveetha Institute of Medical and Technical Sciences, Saveetha University, Chennai, Tamilnadu, India; 2 Department of Oral Pathology and Microbiology, Azeezia College of Dental Sciences and Research, Meyyannoor, Kollam , Kerala, India; 3 Department of Public Health Dentistry, Saveetha Dental College and Hospitals, Saveetha Institute of Medical and Technical Sciences, Saveetha University, Chennai, Tamilnadu, India; 4 Nanobiomedicine Lab, Department of Pharmacology, Saveetha Dental College and Hospitals, Saveetha Institute of Medical and Technical Sciences, Saveetha University, Chennai, Tamilnadu, India; 5 Department of Oral Pathology and Microbiology, Saveetha Dental College and Hospitals, Saveetha Institute of Medical and Technical Sciences, Saveetha University, Chennai, Tamilnadu, India

**Keywords:** Lauric acid, thiocolchicoside, anti-inflammatory agent, spectrophotometer, dose dependant

## Abstract

It is of interest to develop potent and safer anti-inflammatory drugs from plants, as medicinal plants and herbs attained great attention in the medical world due to their multifunctional activities. This article studied the anti-inflammatory effects of lauric acid (LA), thiocolchicoside (TC) and thiocolchicoside-lauric acid (TC-LA) formulation. The anti-inflammatory effects of these compounds were determined by following the methods of inhibition of protein denaturation and proteinase inhibition activity. This was assessed at different concentrations to determine the 50% inhibition concentration (IC50) of the compounds. The result indicated that the activity of LA, TC, TC-LA formulation, and reference drug increased with the increase in the concentration from 10-50 µg/ml, thus proving the activity of LA, TC, and TC-LA formulation against inflammation was in a dose-dependent manner. The percentage of inhibition of protein denaturation was 59.56%, 66.94%, 86.62%, and 60.34% for LA, TC, the combination of TC-LA and standard drug, and the IC50 values were found to be 44.78 µg/mL, 37.65 µg/mL, 27.15 µg/mL and 43.42 µg/mL, respectively. The percentage of proteinase inhibition activity of LA, TC, and a combination of TC-LA and the standard drug was 66.65%, 77.49%, 94.07%, and 69.83%, and IC50 of LA, TC, a combination of TC-LA and standard drug were35.5 µg/mL, 32.12 µg/mL, 24.35 µg/mL and 37.80 µg/mL, respectively. We found out that lauric acid, thiocolchicoside, and thiocolchicoside-lauric acid formulation exhibited significant anti-inflammatory activity.

##  Background:

Inflammation is a condition in which a region of the body develops redness, pain, and swelling as a result of an accident or infection. It is a normal protective mechanism against damage to tissue caused by infection or injuries. Rheumatic diseases, among other inflammatory illnesses [1] are a major cause of mortality worldwide. Nowadays various pain reducing drugs are available to reduce the swelling of the body [2]. Non-steroidal anti-inflammatory drugs (NSAIDs) are mostly used in the management of inflammatory diseases. Some of the currently used drugs are aspirin and other non-steroidal anti-inflammatory drugs. Unfortunately, the administration of these non-steroidal anti-inflammatory drugs (NSAIDs) against inflammation has many side effects such as gastric ulcers, bleeding gastrointestinal tract, and immune system suppression [3]. The most significant drawback of synthetic anti-inflammatory medications currently available is their toxicity and recurrence of symptoms after discontinued treatment [4]. However, many medicinal plants are utilized as effective anti-inflammatory agents due to the presence of secondary metabolites responsible for therapeutic activities. In ancient times, the extract of willow leaves was used to treat inflammation and pain. Anti-inflammatory drugs derived from plants exhibit less or no side effects [5]. Several experiments were done by various researchers using different plants and their parts. Phytochemical constituents from plants are extracted using polar and nonpolar solvents [6; 7]. Some studies revealed that the medicinal property of plant extracts is mainly dependent on the dosage of extract and solvent used for extraction [8; 9]. Nowadays, natural products are found safer than synthetic medications, which are viewed as harmful. So, in search of safety, people are turning towards natural products. About 80% of the medical products of the world depend on bioactive compounds from plants for treating various diseases [10]. The effective alternative pharmacotherapy using plants against inflammation was confirmed by many researchers and pharmaceutical industries. Diverse medicinal plants, richly endowed in the world have been exposed to be effective in the treatment of various diseases including inflammation in traditional medicine [11]. Recently, ginger has been considered effective in curing inflammation and relieving pain proved by Black *et al* [12] and Shah *et al*. [13]. Dried leaves of *M.koenigii* Linn were extracted using water, which caused inhibition of inflammation after administering at different concentrations in male Wistar rats. The acute test result of the aqueous extract of *M.koenigii* showed significant anti-inflammatory activity in a dose-dependent manner as compared to the standard drug. Venkatkumar and Rajeshkumar [14] studied the uses of Mucunapruriens seed extract against inflammation. Moreover, some of the research stated that protein denaturation properties of different plant parts extract such as *Semecarpus Anacardium* bark [15], ethanolic extract of *Wedeliatrilobataon* [16], *F. racemosa* bark powder [17] and Albucasetosaon [18]. Rajesh *et al*. [19] determined the anti-inflammatory activity of the methanolic extract of Niebuhriaapetala by using the methods such as albumin denaturation, antiproteinase action, membrane stabilization, and antilipoxygenase activity. Lauric acid is a saturated fatty acid present in coconut oil and thiocolchicoside is semisynthetic colchicoside derived from plant GloriosaSuperba [20,21].Therefore, it is of interest to examine the anti-inflammatory properties of lauric acid, thiocolchicoside, and TC-LA formulation at different concentrations and determine the 50% inhibition concentration of these compounds.

## Materials and Methods:

Bovine serum albumin (BSA), trypsin, Tris-HCl, lauric acid, thiocolchicoside, perchloric acid, Diclofenac Sodium, casein, dimethyl sulfoxide (DMSO) and hydrochloric acid, were obtained from Sigma Aldrich, Mumbai, India. All the chemicals used in the laboratory were ofanalytical grade (AR).

## Preparation of anti-inflammatory agents:

Lauric acid and thiocolchicoside solutions were prepared separately by mixing LA and TC into 1 ml of methanol and different concentrations of 10µg, 20µg, 30µg, 40µg, and 50µg were prepared. The formulation of thiocolchicoside-lauric acid was prepared by mixing equal concentrations of LA and TC with 1 ml of methanol to determine the enhanced anti-inflammatory activity of LA and TC formulation.

## Inhibition of protein denaturation assay:

The anti-inflammatory activity of lauric acid, thiocolchicoside, and the thiocolchicoside-lauric acid formulation was tested by the following convention method proposed by Muzushima and Kabayashi [22]with specific alterations. A 0.05 mL of lauric acid, thiocolchicoside and thiocolchicoside-lauric acid formulation were taken separately at different concentrations (10µg, 20µg, 30µg, 40µg, and 50µg/mL) and added to 0.45 mL bovine serum albumin (1% aqueous solution) and the pH of the mixture was acclimated to 6.3 utilizing a modest quantity of 1N hydrochloric acid. These samples were incubated at room temperature for 20 min and then heated at 55 °C in a water bath for 30 min. The samples were cooled and the absorbance was estimated spectrophotometrically at 660 nm. Diclofenac Sodium was used as the standard. DMSO was utilized as a control.

The percentage of protein denaturation was determined utilizing the following equation,

% inhibition = ((Absorbance of control - Absorbance of sample)/Absorbance of control) x 100

## Proteinase inhibitory action:

The reaction mixture (2 ml) was prepared by mixing 1 mL of lauric acid at different concentrations (10µg, 20µg, 30µg, 40µg, and 50µg/mL), 0.06 mg trypsin and 1 mL of 20 mM TrisHCl solution (pH 7.4). This 2 ml reaction mixture was incubated for 5 min at 37°C. Then 1% of casein (1 ml) was added and incubated for an additional 20 min. This incubation time was required to inhibit the activity of proteinase. This process was terminated by adding 2 ml perchloric acid (70%) to the reaction mixture, till the formation of a cloudy suspension. Cloudy suspension was subjected to a centrifugation process and the absorbance at 210 nm was measured. Likewise, the experiment on the anti-inflammatory activity of thiocolchicoside and thiocolchicoside lauric acid formulation was done by following the above procedure. TrisHCl was considered blank and Diclofenac Sodium was used as the standard drug. The percentage of inhibition of proteinase inhibitory activity was calculated.

The percentage of protein denaturation was determined utilizing the following equation,

% inhibition = ((Absorbance of control - Absorbance of sample)/Absorbance of control) x 100

## Results:

## Inhibition protein denaturation:

**Figure 1:**
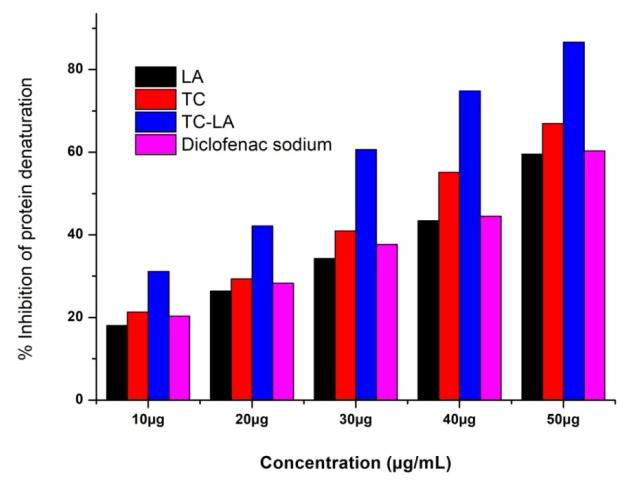
The percentage of protein denaturation inhibition at different concentrations for LA, TC, TC-LA and Diclofenac sodium.

**Figure 2:**
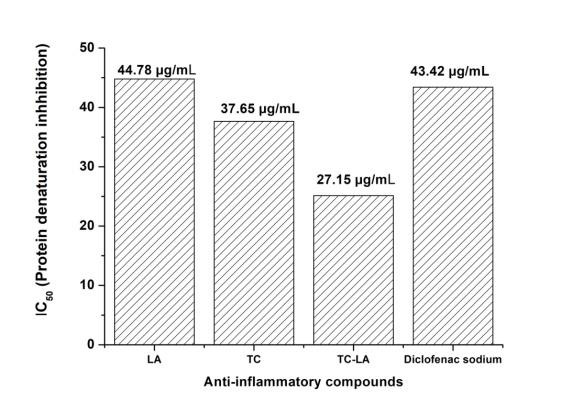
IC50 value of inhibition of protein denaturation for LA, TC, TC-LA and Diclofenac sodium.

Lauric acid, thiocolchicoside, thiocolchicoside-lauric acid formulation, and reference drug were assessed for in vitro anti-inflammatory activity by using inhibition of protein denaturation. Protein denaturation assay using bovine serum albumin is a cheap simple method for examining the anti-inflammatory properties of a drug. LA, TC, and TC-LA combined formulation was able to inhibit protein denaturation, assessed at different concentrations and the result showed that the inhibitory effect of these compounds was concentration-dependent as shown in Figure 1. The highest percentage of protein denaturation inhibition of LA, TC, a combination of TC-LA, and standard drug at the concentration of 50µg/mL were 59.56%, 66.94%, 86.62%, and 60.34%, respectively. The 50% inhibition concentration (IC50) of LA, TC, a combination of TC-LA and standard drug were found to be 44.78 µg/mL, 37.65 µg/mL, 27.15 µg/mL, and 43.42µg/mL, respectively (Figure 2).

## Inhibition of proteinase activity:

**Figure 3:**
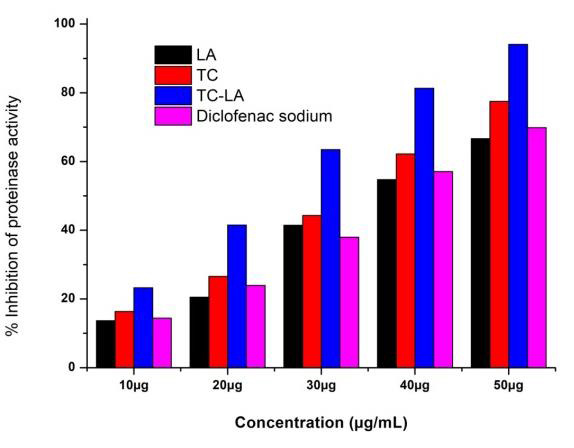
The percentage of proteinase activity inhibition at different concentrations for LA, TC, and TC-LA and Diclofenac sodium.

**Figure 4:**
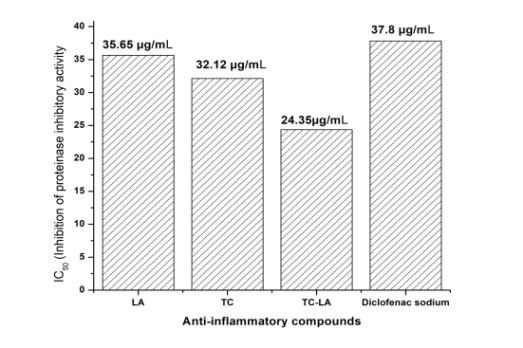
IC50 value of inhibition of proteinase activity for LA, TC, and TC-LA and Diclofenac sodium.

Lauric acid, Thiocolchicoside, Thiocolchicoside-Lauric acid formulation, and Diclofenac sodium (reference drug) exhibited considerable proteinase inhibitory activity observed at different concentrations (Figure 3). The highest percentage of proteinase inhibition activity of LA, TC, TC-LA formulation, and the standard drug was observed at the concentration of 50µg/mL, and the values were found to be 66.65%, 77.49%, 94.07%, and 69.83%, respectively. The 50% inhibition concentration (IC50) of LA, TC, a combination of TC-LA and standard drug were found to be 35.5µg/mL, 32.12 µg/mL, 24.35 µg/mL, and 37.80 µg/mL, respectively (Figure 4, Table 1).

## Discussion:

Plants and plant-derived products are valuable sources of pharmaceutical products due to the presence of secondary metabolites, which have been used widely in the treatment and management of various diseases. Plants are well-known sources for the production of drugs in the pharmaceutical industry. Most of the plants and their compounds are studied for their antioxidant, microbicidal, anticancer, and insecticidal activities [23]. Inflammation is triggered by damaged living tissues resulting from infections caused by bacteria fungi and viruses and also from trauma. And causative factors of inflammation are physical agents and immune responses developed in the body [24]. The main function of inflammation is to prevent and eliminate infectious and harmful agents damaging tissues. An additional function of inflammation is to facilitate the eventual repair of the injured tissues, organs, or systems by removing damaged tissue components. Non-steroidal and corticosteroidal drugs are the commonly prescribed anti-inflammatory drugs for relieving pain. Most of these drugs cause short-term and long-term side effects. There is always a search for natural remedies for the control of pain and thus efforts to produce natural anti-inflammatory drugs have been intensified. Moreover, the development of efficient and safe anti-inflammatory agents is a novel and interesting field for finding alternatives to chemically derived painkillers [25]. Medicinal plants have therapeutic applications due to the presence of phytochemical constituents. The common phytochemical constituents are alkaloids, terpenoids, flavonoids, tannins, phenols, quinines, etc [26]. These phytochemical compounds are responsible for medical applications especially antimicrobial and anti-inflammatory activities [27]. There are various research activities that create evidence for pharmacological applications of plant-based compounds which involve the identification and characterization of bioactive compounds from natural medicinal plants [28,29,30]. Protein denaturation is the main cause of inflammation and the drug used against inflammation is called an anti-inflammatory drug. Most of the drugs are functioning based on dose concentration. Protein denaturation is the loss of activity by biologically important proteins in physical stress or injury [22]. The ability of plant extract to suppress protein denaturation was determined as part of the exploration into the mechanisms underlying the anti-inflammatory mechanism [29]. In this study, three products were examined for their anti-inflammatory activity at different concentrations and compared with a standard drug (Diclofenac sodium). Maximum protein denaturation inhibition of 86.62% was observed at 50µg/ml for TC-LA formulation. It showed enhanced anti-inflammatory activity with the combination formulation of thiocolchicoside-lauric acid than others. Moreover, it also shows that the lowest concentration of 27.15 µg/mL of TC-LA formulation was enough to produce 50% inhibition of protein denaturation. Venkatkumar and Rajeshkumar [14] found 73% protein denaturation inhibition at the concentration of 300 µg/ml of *M. pruriens* seed extract. LA, TC, and TC-LA formulations showed excellent anti-inflammatory activity than the *M. pruriens* seed extract even at low concentrations.

Likewise, maximum inhibition of 94.07% proteinase enzyme activity was found for TC-LA formulation at the highest concentration of 50 µg/ml and the 50% inhibition of TC-LA was noted at 24.35 µg/ml. Venkatkumar and Rajeshkumar [14] found 64% proteinase inhibition at the concentration of 300 µg/ml of *M. pruriens* seed extract. The significance of proteinase inhibition analysis is that the proteinase enzyme is related to conditions like arthritic reactions. They are found in neutrophils and they play a vital role in the formation of tissue damage in inflammatory reactions such as injury, stress, or infections. Rajesh *et al* [19] observed maximum protein denaturation inhibition and anti-proteinase activity by methanolic extract of N.apetala found at 500 µg/ml and the percentage is 78% and 58%, respectively. Huang *et al* [31] investigated the antibacterial and anti-inflammatory effect of capric acid and lauric acid against Propionibacterium acnes which is responsible for causing acne inflammation assessed in mice. The result of the research was both fatty acids significantly reduce IL-6 and IL-8 (IL - Interleukin). Capric acid and lauricacid both actively suppress the secretion of IL-8 and sequentially inhibit phosphorylation of mitogen-activated protein kinase (MAPK) and nuclear factor kappa B (NF-kB) activation. The common mechanism for the inhibition of inflammation is to block cytokines, which inhibit the proliferation of mast cells and also suppress the expression of LPS and COX-2 [31]. When compared to other compounds like LA, TC, and standard drugs, TC-LA exhibited very effective anti-inflammatory activity by following the method of inhibition of protein denaturation and proteinase activity.

## Conclusion:

The pure form of bioactive compounds derived from plants is always considered the best option in the pharmaceutical industry to treat various diseases. The present study investigated the anti-inflammatory activity of lauric acid, thiocolchicoside, and thiocolchicoside - lauric acid formulation, and the inhibition of inflammation was assessed using protein denaturation and proteinase inhibitory action. From the results, it was well understood that three compounds examined in the present study inhibited protein denaturation. Among these three compounds, TC-LA formulation showed enhanced anti-inflammatory activity than standard drugs. From the results obtained, it can be concluded that Thiocolchicoside - Lauric acid formulation can be used as a potent anti-inflammatory drug with less or no side effects. Hence, this study concluded that lauric acid, thiocolchicoside, and a combination of TC-LA may be a good candidate for further invivo studies to develop and design a strong and potent anti-inflammatory agent in the future.

## Figures and Tables

**Table 1:** IC50 value of LA, TC, TC-LA formulation and reference drug against protein denaturation and proteinase activity

**Anti-inflammatory agents**	**50% Inhibition Concentration (IC50)**	
	**Inhibition of protein denaturation (µg/mL)**	**Inhibition of proteinase activity (µg/mL)**
LA	44.78	35.65
TC	37.65	32.12
TC-LA formulation	25.15	24.35
Diclofenac sodium	43.42	37.82
